# Non-seatbelt use and associated factors among Thai drivers during Songkran festival

**DOI:** 10.1186/1471-2458-12-608

**Published:** 2012-08-04

**Authors:** Penprapa Siviroj, Karl Peltzer, Supa Pengpid, Sompong Morarit

**Affiliations:** 1 Department of Community Medicine, Chiang Mai University, Chiang Mai, Thailand; 2 HIV/STI and TB (HAST) Research Programme, Human Sciences Research Council, Pretoria, South Africa; 3 Department of Psychology, University of the Free State, Bloemfontein, South Africa; 4 Department of Health System Management and Policy, University of Limpopo, Medunsa Campus, Pretoria, South Africa; 5 Health Promotion Region 10, Ministry of Public Health, Chiang Mai, Thailand

## Abstract

**Background:**

Road traffic accidents are the second largest cause of burden of disease in Thailand, largely attributable to behavioural risk factors including drinking and driving, speeding, substance abuse and failure to use seatbelts. The aim of this study was to assess the prevalence and associated factors of non-seatbelt use among drivers during Songkran festival in Thailand.

**Methods:**

A cross-sectional survey has been performed to determine the prevalence of seatbelt use among Thai drivers (N=13722) during four days of the Songkran festival. For this sample the population of drivers was consecutively selected from 12 petrol stations in four provinces from each of the four main geographical regions of Thailand. The study was conducted at petrol stations at roads in town, outside town and highway at different time intervals when trained field staff administered a structured questionnaire and performed an observation checklist on seat belt use.

**Results:**

An overall prevalence of 28.4% of non-seatbelt use among drivers was found. In multivariable analysis demographics (being male, younger age, coming from the Northern or Southern region in Thailand), environmental factors (earlier during the Songkran festival, in the morning and late evening and on main roads in town), seatbelt use experiences and attitudes (having been in an accident before, never having used a seatbelt, no intention to use a seatbelt, lack of awareness of danger of non-seatbelt use and lower perceived risk of being caught with non-seatbelt use) and lower exposure to road safety awareness (RSA) campaign (less frequent exposure to RSA campaign, less frequent following of RTI statistics and not talking with others about the RSA campaign) were associated with non-seatbelt use.

**Conclusion:**

Rates of non-seatbelt use by Thai drivers during Songkran festival was 28.4%. Lower exposure to the RSA campaign was found to be associated with non-seatbelt use among drivers during the Songkran festival.

## Background

The Road Traffic Injury (RTI) fatality rate in Thailand was 40 per 100,000 populations, i.e., double the world average for low and middle income countries [1], and RTIs are the second largest cause of burden of disease in Thailand [2]. A number of known behavioural risk factors for RTIs have been identified in Thailand, including drinking and driving, speeding, substance abuse and failure to use seatbelts [3-5]. Aekplakorn et al. [6] conducted an observational study after enactment of the seatbelt law in 1996 in major cities in Thailand. The results showed that 57.3% of motor vehicle drivers did not use seatbelts in January and 69.3% in July 1996. In another study in Thailand non-seatbelt use was found to be considerably lower in passengers than in drivers [5]. Studies on observed seatbelt use in low and middle income countries found high rates of non-seatbelt use among drivers, ranging from 99% among drivers in Kenya [7], 91% in Argentina [8], 83.4% in Ghana [9], 53% in South Africa [10], 50% -32% in Nigeria [11,12], 45% in Russia [13] and 44.1%-32.7% in China [14,15]. In most of these studies passengers in motor vehicles seemed to use less often a seatbelt than drivers [5,9,11,14]. Factors associated with non-seatbelt use among drivers were male gender [6,9,16], younger age [9], professional and pickup versus general drivers [5,9,14], lowest within the Central Business District (CBD) compared to the outskirts of the city [6,9], lower on highways than on local streets [17], lower on urban roads compared to those on main highways and rural roads [13], lower at daytime, early in the morning than at night [14,15], and fatalistic orientation [10].

Songkran is the New Year celebration in Thailand, set by the solar calendar since ancient times. It takes place between 13 and 15 April. At Songkran festival are major holidays that encourage a million of travellers who travel to/from their hometown and doing the activities during these holiday periods [18]. Unfortunately, number of road accidents, fatalities, and injuries, increase dramatically; in April the number of road traffic fatalities almost 1200, way above average of <1000 [18]. The daily fatalities during Songkran festival rise up to 84 and 95 persons per day, an increase of 147% and 179%, respectively, compared with an average of 34 persons per day in the non-festival period. Similarly, daily injuries during Songkran holidays increased to 4,900 and 5,650 persons, compared with an average of 2,468 persons per day during the non-festival period [19,20]. The risks of road traffic accidents during long holidays such as New Year and Songkran festival were found to be alcohol drinking driver, high speed drivers and not using safety equipments [20]. In Thailand there is law on seatbelt wearing; according to the Road Traffic Act 1979 a seatbelt must be fastened at all time during driving and passengers are also obliged to fasten the seatbelt at all times [21]. From 1997 an active public education programme was undertaken on a national scale to raise awareness about road safety and to support law enforcement. This included dissemination of knowledge through multiple channels, e.g., roadside posters, stickers on the back of vehicles, sporadic radio and TV programmes or spots, public announcements and press reports [22]. After 2000, communication about the law was increased and both governmental and nongovernmental agencies started to participate in traffic injury prevention and control programmes including seatbelt wearing among drivers [23,24]. This included also increased road safety awareness (RSA) campaigns during the Songkran festival [21], but seemingly not everywhere the full range of RSA campaigns was implemented [25]. Among the risks of road traffic accidents during long holidays the lack of using safety equipments has not been adequately studied. Therefore, the aim of this study was to assess the prevalence and associated factors of non-seatbelt use among drivers during Songkran festival in Thailand.

## Methods

### Sample and procedure

A cross-sectional survey has been performed to determine the prevalence of helmet use among drivers. The recruitment period of this project was during four days of the Songkran festival from 13–16 April 2007. For this sample the population of drivers from 12 petrol stations were selected from four provinces from each of the four main geographical regions of Thailand excluding Bangkok. Provinces were Chiang Mai, Lampang, Nakhon Sawan and Phichit in the northern region, Nakhon Ratchasima, Khon Kaen, Udon Thani, and Loei in the Northeastern region, Songkhla, Phuket, Surat Thani, and Trang in the southern region, and Phra Nakhon Si Ayutthaya, Chonburi, Chachoengsao, and Phetchaburi in the central region. In total 48 petrol stations (three petrol stations per province) was selected using quota sampling. In town, the petrol station on the road with the largest shopping mall was selected; out of town the petrol station on the road leading to the largest district was selected; in terms of petrol station on the highway, each province only has one highway. If there was more than one petrol station on the selected road or highway, the largest petrol station was selected. The study team spent four days at each petrol station road venue (roads in town, outside town and highway) from 7:00–9:00, 13:00–15:00, 17:00–19:00, 22:00–24:00. All consecutive motor vehicle occupants who entered the petrol station were asked to participate by trained personnel (who were students from Chiang Mai University that were trained by the research team) while they were having their gas tank filled. The number of vehicles and time interval for vehicle selection were determined by the availability of field staff to conduct a motor cycle rider observation, interview and alcohol test. The target sample size was 100 drivers from each of the petrol stations per time period, except during 22:00–24:00 for which 50 drivers were targeted. Trained field staff administered a structured questionnaire and performed an observation checklist. The project was approved by the Ethics Committee for research in human subjects of the public health programme, Chiang Mai University.

### Measures

The primary outcome of the study was seatbelt use. Seatbelt use was assessed by observation. The questionnaire covered demographic data, vehicle characteristics, history of road traffic accidents, known risk factors such as, age, sex, environmental factors, seatbelt use experiences and attitudes, and exposure to the road safety awareness (RSA) campaign.

### Data analysis

Data were analyzed using Statistical Package for the Social Sciences (SPSS) for Windows software application programme version 19.0. Frequencies, means, standard deviations, were calculated to describe the sample. Data were checked for normality distribution and outliers. For non-normal distribution non-parametric tests were used. Associations of non-seatbelt use were identified using logistic regression analyses. Following each univariate regression, multivariable regression models were constructed. Independent variables from the univariate analyses were entered into the multivariable model if significant at P<0.05 level. For each model, the R^2^ are presented to describe the amount of variance explained by the multivariable model. Probability below 0.05 was regarded as statistically significant.

## Results

### Sample characteristics

The total sample included 13722 drivers (288 refused, response rate 98.3%); 77.4% of the drivers were male and 22.6% female. The majority of the drivers (79.9%) were between 26 to 59 years old and about half (50.7%) were driving a pickup. Driver participation in the study was equally distributed across four of Thailand’s four regions, four data collection times during the day, four dates of data collection and three locations of data collection. The overall prevalence of non-seatbelt use was 28.4% (see Table 1). Seatbelt use of passengers was also assessed. In 33.2% of the cases or cars there was no passenger, and in 66.8% of the cars where there was a passenger 60.3% were not and 39.7% were wearing a seatbelt. More female (67.2%) than male (50.6%) passengers had not been wearing a seatbelt.

**Table 1:** Sample characteristics of drivers during Songkran festival

	**Total**		**Non-seatbelt use of driver**	
	**N**	**%**	**N**	**%**
All	13722		3879	28.4
Male	10603	77.4	3160	29.9
Female	3095	22.6	719	23.3
Age (by self-report)				
<18	180	1.3	85	47.5
18–25	2379	17.4	803	33.8
26–59	10950	79.9	2922	26.8
60 or more	191	1.4	63	33.2
*Type of car*				
Mini-truck	6948	50.7	2157	31.1
Saloon	5416	39.6	1326	24.5
Mini bus	965	7.0	209	21.7
Truck	365	2.7	179	49.0
*Region*				
North	3575	26.1	1196	33.5
Central	3455	25.2	1057	30.2
Northeast	3333	24.3	427	14.4
South	3359	24.5	1151	34.3
*Data collection time*				
07.00-09.00	3897	28.4	1114	28.7
13.00-15.00	3914	28.5	1158	29.7
17.00-19.00	3918	28.6	1019	26.1
22.00-24.00	1993	14.5	530	29.7
*Date of data collection*				
13 April 2007	3401	24.8	1065	31.5
14 April	3435	25.0	1030	30.0
15 April	3442	25.1	860	25.0
16 April 2007	3444	24.1	926	27.0
*Location of data collection*				
Main road in town	4677	34.1	1569	33.7
Roads out of town	4623	33.7	1333	28.9
Highway	4422	32.2	979	22.2

### Seatbelt use experiences, attitudes and road safety awareness campaign exposure

Regarding previous driving experience, 25.6% of the sample indicated that they had been in an accident before. Of those who had ever been in an accident before, most had been involved in the accident as a driver (77.5%), followed by passenger (22.5%) and pedestrian (2.0%). A large group of participants (46.6%) indicated that they had not usually been using a seatbelt before and 41.5% had not intended to use a seatbelt. The majority (73.7%) perceived a danger of not wearing a seatbelt and 53.0% were highly aware of the danger of not wearing a seatbelt. A significant number of 26.4% indicated that they had been caught by the police because of not wearing a seatbelt and 67.3% perceived a moderate to high risk about being caught by the police because of not wearing a seatbelt. Almost all (90.4%) had heard about the RSA campaign and more than one-thirds (36.3%) had frequently heard or seen the RSA campaign on the radio or on TV. More than half (57.0%) of the participants had been talking to others about the RSA campaign. One-thirds (33.3%) liked the RSA campaign very much, 31.4% frequently followed the TV news reports on road traffic injury (RTI) statistics and more than half (54.7%) believed perceived that the RSA campaign had a high effect (see Table 2).

**Table 2:** Seatbelt use experiences, attitudes and exposure to road safety awareness campaign of drivers during Songkran festival

**Variables**	**Response options**	**Total**		**Non-seatbelt use of driver**	
				**N**	**%**
**Seatbelt use experiences and attitudes**					
Been in accident before	No	10123	74.4	2726	27.0
	Yes	3482	25.6	1104	31.8
Driver status when in accident	Driver	2603	75.5	812	31.3
	Passenger	775	22.5	242	31.2
	Pedestrian	69	2.0	28	40.6
Not usually used a seatbelt before	No	6372	46.6	2303	36.3
	Yes	7310	53.4	1567	21.5
Intention to use a seatbelt	No	5662	41.5	1777	31.5
	Yes	7978	58.5	2081	26.2
Awareness of danger of no seatbelt use	Low	652	4.8	270	41.4
	Moderate	5769	42.2	1756	30.5
	High	7243	53.0	1841	25.5
Perceived risk about being caught by the police because of not wearing a seatbelt	No risk	1961	14.4	553	28.2
	Low risk	2494	18.3	821	32.9
	Moderate risk	5209	38.2	1367	26.3
	High risk	3975	29.1	1122	28.4
Caught not wearing a seatbelt	No	10045	73.6	2885	28.8
	Yes	3608	26.4	978	27.3
**Exposure to road safety awareness (RSA) campaign**					
Heard of RSA campaign	No	1312	9.6	396	30.3
	Yes	12410	90.4	3485	28.2
Frequency of exposure to RSA campaign	Never	1059	7.8	365	34.5
	Not often	7342	54.1	2048	28.0
	Frequently	4928	36.3	1374	27.9
	Not sure	235	1.7	68	28.9
Talking to others about RSA campaign	Never	3878	28.3	1316	34.0
	Ever	7795	57.0	1999	25.7
	Not sure	2014	14.7	553	27.5
Follows TV news on RTI statistics	Never	1288	9.4	561	43.7
	Not often	7569	55.4	2149	28.5
	Frequently	4287	31.4	1024	24.0
	Not sure	514	3.8	129	25.3
How feels about RSA campaign	Not like	952	7.0	332	34.9
	Like a little bit	7566	55.2	2324	30.8
	Like very much	4567	33.3	1047	23.0
	Not sure	610	4.5	162	26.6
Perceived effect of RSA campaign	Low	1492	10.9	450	30.4
	Medium	4700	34.4	1621	34.6
	High	7473	54.7	1798	24.1

### Association between non-seatbelt use and demographics, experiences, attitudes and RSA campaign exposure

In multivariable analysis demographics (being male, younger age, coming from the Northern or Southern region in Thailand), environmental factors (earlier during the Songkran festival, in the morning and late evening and on main roads in town), seatbelt use experiences and attitudes (having been in an accident before, not usually using a seatbelt, no intention to use a seatbelt, lack of awareness of danger of non-seatbelt use and lower perceived risk of being caught with non-seatbelt use) and lower exposure to RSA campaign (less frequent exposure to RSA campaign, less frequent following of RTI statistics and not talking with others about the RSA campaign) were associated with non-seatbelt use (see Table 3).

**Table 3:** Association between non-seatbelt use and demographics, environmental factors, seatbelt use experiences and attitudes and RSA campaign exposure (during Songkran festival)

	**Variables**	**Unadjusted Odds Ratio**	**Adjusted Odds Ratio**^**a**^
**Demographics**	Female vs. Male	1.41 (1.28-1.54)***	1.19 (1.06-1.34)**
	*Age*		
	<18 years	1.00	1.00
	18–25	0.57 (0.42-0.77)***	0.79 (0.54-1.14)
	26–59	0.40 (0.30-0.54)***	0.65 (0.45-0.93)*
	60 or more	0.55 (0.36-0.84)***	0.76 (0.46-1.26)
	*Region*		
	North	1.00	1.00
	Central	0.88 (0.80-0.97)*	0.75 (0.67-0.85)***
	Northeast	0.34 (0.30-0.38)***	0.27 (0.23-0.31)***
	South	1.04 (0.94-1.15)	1.01 (0.90-1.21)
**Environmental factors**	Mini-truck (Pickup)	1.00	1.00
	Saloon	0.72 (0.66-0.78)***	0.70 (0.63-0.77)***
	Mini bus	0.61 (0.52-0.72)***	0.53 (0.44-0.64)***
	Truck	2.13 (1.72-2.65)***	1.91 (1.48-2.46)***
	*Day of Songkran festival*		
	13 April 2007	1.00	1.00
	14 April	0.94 (0.84-1.04)	0.95 (0.84-1.07)
	15 April	0.73 (0.65-0.81)***	0.77 (0.68-0.87)***
	16 April 2007	0.80 (0.72-0.89)***	0.80 (0.71-0.91)***
	*Time of the day*		
	07.00-09.00	1.00	1.00
	13.00-15.00	1.05 (0.95-1.16)	1.04 (0.92-1.16)
	17.00-19.00	0.88 (0.80-0.97)*	0.87 (0.78-0.98)*
	22.00-24.00	1.05 (0.93-1.18)	1.05 (0.91-1.20)
	*Type of road*		
	Main road in town	1.00	1.00
	Roads out of town	0.80 (0.73-0.88)***	0.70 (0.63-0.78)***
	Highway	0.56 (0.51-0.62)***	0.52 (0.47-0.59)***
**Seatbelt use experiences and attitudes**	Been in accident before	1.26 (1.16-1.37)***	1.18 (1.07-1.30)***
	Driver status when in accident		
	Driver	1.00	---
	Passenger	1.00 (0.84-1.19)	
	Pedestrian	1.50 (0.92-2.45)	
	Not usually used a seatbelt	2.08 (1.93-2.25)***	2.40 (2.19-2.63)***
	No intention to use a seatbelt	1.30 (1.20-1.40)***	1.28 (1.17-1.41)**
	*Awareness of danger of no seatbelt use*		
	High	1.00	1.00
	Moderate	1.29 (1.19-1.39)***	1.47 (1.34-1.62)***
	Low	2.07 (1.75-2.44)***	1.55 (1.28-1.89)***
	*Perceived risk to be caught with no seatbelt use*		
	High	1.00	1.00
	Moderate	0.90 (0.82-0.99)*	0.82 (0.73-0.92)***
	No/low	1.13 (1.03-1.24)*	0.97 (0.86-1.09)
	Caught not wearing a seatbelt	0.93 (0.85-1.01)	---
**Exposure to road safety awareness campaign**	Not heard RSA campaign	1.11 (0.98-1.25)	---
	*Frequency of exposure to RSA campaign*		
	Frequently	1.00	1.00
	Not often	1.00 (0.93-1.10)	1.08 (0.97-1.19)
	Never/not sure	1.30 (1.14-1.48)***	1.92 (1.82-2.29)***
	Not talking to others about RSA campaign	1.49 (1.37-1.62)***	1.14 (1.04-1.26)**
	*Follows RTI stats*		
	Frequently	1.00	1.00
	Not often	1.26 (1.16-1.38)***	1.18 (1.06-1.31)**
	Never, not sure	1.99 (1.77-2.24)***	1.72 (1.48-1.99)***

^a^**Hosmer & Lemeshow Chi-square=16.48, P=0.036; Nagelkerke R**^**2**^**: 0.17.**

## Discussion

In this study among a large sample of divers in Thailand 28.4% were observed of non-seatbelt use, which seemed to be better than in previous studies in Thailand [6]. Previous studies of non-seatbelt use among drivers in low and middle income countries seemed to have also found worse rates of non-seatbelt use than in the current study [7-15]. In concordance with other studies, this study found that being male [6,9,16], younger age [9], professional and pickup versus general drivers [5,9,14,26,27], location of road (main roads in town) [6,9], time of the day (earlier time in the day) [14,15] were associated with non-seatbelt use among drivers. The study also found in concordance with most studies [5,9,11,14] that passengers in motor vehicles had used less often a seatbelt than drivers. Non-seatbelt use was in this study higher at the beginning than at the end of Songkran festival and it was found higher when driving on main roads in town than out of town or on the highway. Some of these differences may be explained by the actual driving location, as it could be that higher non-seatbelt use was found when celebrating the Songkran festival in their home town involving higher non-seatbelt use compared to celebrating the Songkran festival away from current residence which involves driving on the high way and possibly less non-seatbelt use. Among truck drivers non-seatbelt use was found to be higher than among drivers of a saloon car or minibus, which may be explained by different personalities. It is recommended that the RSA campaign should be improved by specifically targeting risk groups such as truck drivers and risky places such as main road in town.

Further, having been in an accident before, not usually having used a seatbelt, not having intended to use a seatbelt, lack of awareness of the danger of non-seatbelt use and lower perceived risk of being caught with non-seatbelt use was found in this study to be associated with non-seatbelt use. Drivers may seem not to be inclined to protect themselves voluntarily against very low probability threats [28]. Thai people also believe in karma, meaning that if the time for an accident or death has come one cannot avoid it.

Importantly, lower exposure to RSA campaign (less frequent exposure to RSA campaign, less frequent following of RTI statistics and not talking with others about the RSA campaign) were in this study associated with non-seatbelt use. Phillips et al. [29] found from a meta-analysis of 67 studies that the weighted average effect of road safety campaigns was a 9% reduction in accidents.

### Study limitations

Caution should be taken when interpreting the results of this study because of certain limitations. As this was a cross-sectional study, causality between the compared variables cannot be concluded. A further limitation was that some variables were assessed by self-report and desirable responses may have been given. Other examples of limitations include that other substance use (illicit drugs) were not assessed, as found to be prevalent in other studies in Thailand [19]. Future studies should also investigate non-helmet use among motorcyclists in Thailand, as it has been found to be a significant problem in previous studies [21].

## Conclusion

Rates of non-seatbelt use by Thai drivers and passengers during Songkran festival was 28.4%. Lower exposure to the RSA campaign was found to be associated with non-seatbelt use among drivers during the Songkran festival.

## Competing interests

The authors declare that they have no competing interests.

## Authors’ contributions

PS, KP and SP were the main contributors to the conceptualization of the study. KP, PS and SP contributed significantly to the first draft of the paper and all authors contributed to the subsequent drafts and finalization. All authors read and approved the final manuscript.

## Pre-publication history

The pre-publication history for this paper can be accessed here:

http://www.biomedcentral.com/1471-2458/12/608/prepub
